# Radiodermatitis: severity, predictive factors and discontinuation of radiotherapy in patients with anal and rectal cancer

**DOI:** 10.1590/1980-220X-REEUSP-2021-0378en

**Published:** 2022-06-15

**Authors:** Larissa Jucá Dantas Bastos, Regina Serrão Lanzillotti, Marcos Antônio Gomes Brandão, Rafael Celestino da Silva, Fabiana Verdan Simões

**Affiliations:** 1 Instituto Nacional de Câncer José Alencar Gomes da Silva, Programa de Residência Multiprofissional em Oncologia, Rio de Janeiro, RJ, Brazil.; 2 Universidade do Estado do Rio de Janeiro, Rio de Janeiro, RJ, Brazil.; 3 Universidade Federal do Rio de Janeiro, Escola de Enfermagem Anna Nery, Departamento de Enfermagem Fundamental, Rio de Janeiro, RJ, Brazil.

**Keywords:** Radiodermatitis, Rectal Neoplasms, Prevalence, Nursing Care, Radiodermatitis, Neoplasias del Recto, Prevalencia, Atención de Enfermería, Radiodermatite, Neoplasias Retais, Prevalência, Cuidados de Enfermagem

## Abstract

**Objective:**

to determine the prevalence of radiodermatitis, severity grades and predictive factors of its occurrence in patients with anal and rectal cancer followed up by the nursing consultation, and to analyze the association of severity grades of radiodermatitis with temporary radiotherapy interruption.

**Method:**

a quantitative, cross-sectional and retrospective study, carried out with 112 medical records of patients with anal and rectal cancer undergoing curative radiotherapy followed up in the nursing consultation. Data were collected using a form and analyzed using analytical and inferential statistics.

**Results:**

99.1% of patients had radiodermatitis, 34.8% of which were severe. The predictive factors were female sex, age greater than 65 years, anal canal tumor, treatment with cobalt device and IMRT technique. Treatment interruption occurred in 13% of patients, associated with severe radiodermatitis.

**Conclusion:**

there was a high prevalence of radiodermatitis, mainly severe, which resulted in treatment interruption.

## RESUMO

**Objetivo:**

determinar a prevalência da radiodermatite, os graus de severidade e os fatores preditivos da sua ocorrência em pacientes com câncer de canal anal e reto acompanhados pela consulta de enfermagem, e analisar a associação dos graus de severidade da radiodermatite com a interrupção temporária da radioterapia.

**Método:**

pesquisa quantitativa, seccional e retrospectiva, realizada com 112 prontuários de pacientes com câncer de canal anal e reto submetidos à radioterapia curativa acompanhados na consulta de enfermagem. Dados foram coletados por formulário e analisados empregando-se estatística analítica e inferencial.

**Resultados:**

99,1% dos pacientes apresentaram radiodermatite, sendo 34,8% graus severos. Os fatores preditivos foram sexo feminino, idade maior que 65 anos, tumor de canal anal, tratamento com aparelho de cobalto e técnica IMRT. A interrupção do tratamento ocorreu em 13% dos pacientes, associada à radiodermatite severa.

**Conclusão:**

houve alta prevalência de radiodermatite, principalmente grau severo, que resultou em interrupção do tratamento.

## RESUMEN

**Objetivo:**

determinar la prevalencia de radiodermatitis, los grados de severidad y los factores predictores de su ocurrencia en pacientes con cáncer anal y rectal seguidos de la consulta de enfermería, y analizar la asociación de los grados de severidad de la radiodermatitis con la interrupción temporal de radioterapia.

**Método:**

investigación cuantitativa, transversal y retrospectiva, realizada con 112 prontuarios de pacientes con cáncer anal y rectal en tratamiento con radioterapia curativa seguidos en la consulta de enfermería. Los datos fueron recolectados mediante un formulario y analizados mediante estadística analítica e inferencial.

**Resultados:**

el 99,1% de los pacientes presentaban radiodermatitis, de las cuales el 34,8% eran graves. Los factores predictores fueron sexo femenino, edad mayor de 65 años, tumor del canal anal, tratamiento con aparato de cobalto y técnica de IMRT. La interrupción del tratamiento se produjo en el 13% de los pacientes, asociada a radiodermitis grave.

**Conclusión:**

hubo una alta prevalencia de radiodermatitis, principalmente severa, lo que obligó a la interrupción del tratamiento.

## INTRODUCTION

Anal canal carcinoma is an infrequent neoplasm that corresponds to 2.6% of all malignant tumors of the digestive system. In 2019, it had an estimated 8,300 new cases and 1,280 deaths in the United States^(1,2)^. In Brazil, epidemiological data on this neoplasm are not clearly known. On the other hand, epidemiological data on rectal cancer are found in the context of colorectal cancer, which covers both sites. The Brazilian National Cancer Institute estimated, for 2020, the incidence of 9% of colorectal cancer and 41,010 new cases for the 2020–2022 triennium, corresponding to the third most common neoplasm among men and the second among women^(3)^.

The standard treatment for anal canal cancers consists of pelvic radiotherapy (RT) associated with 5-fluorouracil and mitomycin infusion^(4)^. In rectal cancer, from stage II, combined treatment including chemotherapy (CT), RT and surgery is indicated in order to obtain better sphincter control and decrease local recurrence^(5)^.

RT is an important modality for cancer treatment, however, even with advances in radiation techniques and devices, patients still experience undesirable events that compromise quality of life. Among these are radiodermatitis, which is a set of skin changes that occur from internal to external structures^(6–8)^.

It is classified as acute when toxicity arises during treatment or up to three months after completion, characterized by mild to severe erythema, dry desquamation, wet desquamation and, in more severe cases, ulceration, hemorrhage and tissue necrosis may occur. It is classified as chronic radiodermatitis when it appears three months after the end of treatment, and its symptoms are ischemia, pigmentary changes, thickening, telangiectasia, ulceration and fibrosis^(7,8)^.

The grade of toxicity, according to the acute radiation morbidity score criterion of the Radiation Therapy Oncology Group (RTOG) scale, is classified as: Grade 0: no change from baseline; Grade 1: follicular erythema, weak or dull, epilation and/or dry scaling, decreased sweating; Grade 2: painful or shiny erythema, localized moist desquamation and/or moderate swelling; Grade 3: confluent moist desquamation and/or significant edema; Grade 4: ulceration, hemorrhage and necrosis; Grade 5: effect that causes death^(9)^.

Another commonly used scale is the Common Terminology Criteria for Adverse Events (CTCAE)^(10)^. In this scale, radiodermatitis is considered an adverse event, i.e., an unfavorable event and an unintended sign associated with the use of medical treatment. Toxicity is classified as: Grade 1: weak erythema or dry desquamation; Grade 2: moderate to severe erythema, patchy moist desquamation, especially in skin folds, moderate swelling; Grade 3: wet scaling in areas other than the folds, bleeding induced by minor trauma or abrasion; Grade 4: skin necrosis or dermal ulceration, spontaneous bleeding from the involved site; Grade 5: death^(10)^.

In both scales, the classification of toxicity represents grade severity of radiodermatitis, namely: Grade 0: without radiodermatitis; Grade 1: mild radiodermatitis; Grade 2: moderate; Grade 3: severe; Grade 4: life-threatening; Grade 5: death linked to radiodermatitis^(10)^.

In general, the phenomenon of radiodermatitis stands out for its magnitude, which is identified by the high prevalence. Evidence indicates that about 93% to 99% of cancer patients, at different sites, under RT treatment with curative indication, develop this event^(11,12)^.

In patients with anal and rectal cancer, the prevalence of radiodermatitis is unclear, although there is a greater propensity for its occurrence in this site, explained by variables such as folds in the region and risk of constant humidity and friction, CT treatment protocols and the toxicity of RT in the lower gastrointestinal system, which causes episodes of diarrhea^(13)^. Studies that assessed the therapy in these patients prioritized the information only of severe adverse events, Grade 3 and 4 and, eventually, Grade 2, an aspect that hinders the knowledge about the prevalence of radiodermatitis^(14,15)^.

Radiodermatitis management integrates the care provided by nurses through the nursing consultation in RT. From the nursing diagnostic point of view, physical examination focused on the irradiated area can identify evidence of skin toxicity^(16)^. Among the indicators to be analyzed are the predictive factors of radiodermatitis outcome, which include clinical, sociodemographic and treatment variables^(7,8,11,12)^.

In general, the guidelines that address radiodermatitis bring the predictive factors for this outcome in a generic way, i.e., they do not present such predictive factors according to the specificity of each type of cancer, as shown in some studies^(12,17)^.

In a study developed with 167 patients with head and neck cancer treated with RT, treatment variables, such as type of device and technique, and clinical variables, such as the presence of comorbidities, were associated with cases of severe radiodermatitis^(12)^. In research with 117 patients with breast cancer submitted to RT 3D, there was an association of the development of radiodermatitis with higher doses of radiation and use of daily bolus^(17)^.

Therefore, the knowledge of these predictive factors in the cases of patients with anal and rectal cancer is essential for nurses’ clinical judgment and the choice of the best evidence-based nursing interventions, with a view to managing toxic skin reactions.

Such interventions are extremely relevant to minimize the impact of radiodermatitis on prognosis, since, depending on the extent of the injury, treatment interruptions may occur. An example can be seen in the study of patients with head and neck cancer, which assessed the association of cases of severe radiodermatitis with treatment interruption. Of the 19 (11%) patients who presented Grade 3, 53% had temporary discontinuation of treatment, with an average of 11 days of interruption^(12)^. Furthermore, unplanned interruptions during RT may reduce cure rates of cancer in regions such as head and neck, cervix, breast, lung, and anus^(18,19)^.

Based on the above, the following research question was raised: what is the prevalence, severity grades and predictive factors of radiodermatitis in patients with anal and rectal cancer followed by a nursing consultation and what association exists between grade severity of radiodermatitis and the temporary RT interruption?

The objectives were to determine the prevalence of radiodermatitis, grade severity and the predictive factors of its occurrence in patients with anal and rectal cancer followed by the nursing consultation, and to analyze the association of grade severity of radiodermatitis with the temporary RT interruption.

## METHOD

### Design of Study

This is quantitative, cross-sectional and retrospective research, carried out through documentary analysis of medical records of patients with anal and rectal cancer.

### Local

The research was developed in the RT outpatient clinic of an institution specialized in cancer located in the city of Rio de Janeiro. In this institution, patients are treated at the RT service from the appointment through the State Regulation System. Patients’ enrollment is classified as a patient coming from the institution sector itself, or referred from a public external health institution. Six RT equipment and a multidisciplinary team composed of nurses, physicians, physicists, nursing technicians and RT technicians are available.

After scheduling a medical consultation, the following procedures are adopted to start treatment: on the day of the first consultation, a radio-oncologist analyzes the RT (curative or palliative) intention, prescribes the dose and chooses the device. Patients and family members receive guidance on the procedure, side effects, expected benefit, and informed consent is obtained from patients. Subsequently, two procedures are scheduled for patients before RT start: tomography, which is performed with the objective of acquiring images for RT planning; and simulation, which aims to determine the treatment position, target volume, field geometry, among other relevant information. Applications are expected to start on the day of simulation or up to one week after.

When patients initiate ionizing radiation applications, they are referred by a radio-oncologist to participate in the “Radiated Skin Care Guidance Group”; from the insertion in this group, they are scheduled for individual follow-up in the nursing consultation, in which interventions are prescribed according to the institution’s Care Protocol for Radiodermatitis^(20)^.

The criteria for weekly follow-up in the nursing consultation of patients with anal canal and rectum cancer are: patients undergoing treatment with a total dose of 2,000 centigrays (cGy); and number of applications of ionizing radiation greater than ten fractions. The other patients are treated at the consultation without regular follow-up.

### Selection Criteria

Patients diagnosed with anal and rectum canal cancer, undergoing curative RT, with or without dose increase, accompanied by at least three nursing consultations were included. Patients who were not registered in the medical record on skin conditions according to the RTOG scale until the end of treatment and who had their RT replanned by a radio-oncologist throughout treatment were excluded.

### Population

The definition of the number of participants considered patients with anal and rectal cancer treated in the RT sector, surveyed from January to December 2017. The total number of patients registered in the institution’s computerized system during this research period and who met the inclusion criteria was 140 patients. Of these, after analyzing the exclusion criteria, 28 records were discarded, with the final number of 112 patients participating in the study.

The choice of the year 2017 is justified, as this research is an excerpt of a macro-project on radiodermatitis, developed within the scope of the activities to conclude the institution’s Multidisciplinary Residency. Therefore, due to time limitations, we opted for the records of the period of one year, in order to enable data collection.

### Data Collection

Secondary data were collected, from August to December 2018, through access to patients’ physical records in the chosen institution’s archive service. After project approval by the institutional Ethics Committee and with the list of potential patients identified, medical records were requested one day before collection at the hospital’s archive service, which released ten records per day.

Structured form developed for data collection from an integrated research project on radiodermatitis in cancer patients in different sites, coordinated by one of the authors of this article, was licensed. This form has already been tested in a section of this project, developed with patients with head and neck cancer^(12)^.

The variables were chosen considering the existing evidence on risk factors for radiodermatitis, as well as the guidelines on the subject of the Oncology Nurses Society^(6–8)^. In the particularity of this research, the form was adapted considering the investigated population’s specificities, contemplating sociodemographic (age, sex, skin color, education, marital status, consensual coexistence, lifestyle habits (alcoholism and smoking), clinical (comorbidities, stage and tumor site) and treatment variables (treatment device, grade of radiodermatitis according to RTOG classification, chemotherapy treatment concomitant with RT treatment, temporary RT treatment interruption and number of days interrupted, data on treatment technique, applied dose and dose fractioning).

Data were collected by the main researcher in the following medical record sections: nurses’ records, which contained information on focused physical examination of the skin in the irradiated area, patient complaints and care prescriptions; medical records, with information on treatment progress and interruptions; and RT planning form, which included information on the treatment technique and dose applied. In this last section, data collection was performed by a physicist from the institution’s RT sector, who received information about the research and training on how to fill out the form.

### Data Analysis and Treatment

After the collection phase, the database was built with the aid of software SPSS, version 23, which was revised and, subsequently, data were tabulated. This tabulation occurred with the construction of contingency tables, from which statistical analyses were performed. Thus, descriptive, analytical and inferential statistics were used.

Descriptive statistics was applied to describe the profile of subjects in relation to clinical and sociodemographic variables, with simple frequency and percentage. Age was analyzed according to measures of central tendency (mean, mode, median) and measure of variability (standard deviation).

The prevalence rate of radiodermatitis was calculated from the number of individuals affected by radiodermatitis, divided by the total number of people and multiplied by 100. Subsequently, the clinical and sociodemographic variables were crossed to analyze the association of these predictive factors with the radiodermatitis outcome. The association analysis was based on the *Odds Ratio* and Pearson’s chi-square test, adopting a significance level of 5%. The *Odds Ratio* confidence interval estimate corresponded to 95% reliability.

In the analysis process, the variables were dichotomized, since we opted for inference according to the binomial distribution of radiodermatitis severity. Thus, as the toxicity classification of the RTOG scale was applied and one of the research objectives included the analysis of the impacts of the most severe grades of radiodermatitis on treatment interruption, Grade 0 (non-observation of the event) with severity 1 and 2, due to being considered less severe grades (mild and moderate), as well as agglutinating Grade 3 and 4 (severe and life-threatening) were included^(10)^. As for the clinical variables, in the strata related to the tumor site, anal margin was included in the anal canal stratum and straighter anal canal in the stratum rectum, due to the lower percentages found in these sites.

Regarding the sociodemographic variables, the schooling variable was dichotomized, in which the illiterate and those who were barely literate were considered as “Uneducated”, and those with elementary, high school and higher education, as “Educated”. In skin color, the subdivision was made between white and non-white (brown, yellow and black), due to self-declaration. Marital status was also categorized in conjugal coexistence (married) and without conjugal coexistence (single, widowed and divorced).

In some crosses assessing the association between variables and radiodermatitis grade, the number of participants was lower than the final quantitative of this research (112 patients), because no data were found in medical records, such as comorbidity (108), alcoholism (106), smoking (106) and combined CT (109).

### Ethical Aspects

The research project was approved by the Research Ethics Committee of the studied institution, under Opinion 2756072, in 2018, in accordance with Resolution 466/2012. As this was a documentary study, the Informed Consent Form was waived.

## RESULTS

Patients enrolled in the RT sector from other sectors of the studied institution corresponded to 69.6% of participants, and those from external institutions, to 30.4%. The mean group age was 63.7 years, with a standard deviation of 12.98. The minimum age was 32 years, and the maximum was 98 years. The most representative modal group age was 70 years, indicating that the group tended to more advanced ages. Regarding the median, it corresponded to 64 years.

Regarding sex, there was a predominance of females, with 59.8%. In skin color, the non-white group showed a relative frequency of 60.8%. Regarding education, the “Educated” had a frequency of 95.5%, of which 45.5% attended elementary school, 40.2%, high school, and 9.8%, higher education. When considering marital status, those without marital cohabitation were 54.5%, while those married corresponded to 45.5%. Regarding alcohol consumption and smoking, 106 individuals were assessed. Smokers and former smokers corresponded to approximately 46% of the sample, and alcoholics, to 42%.

Among the clinical variables, at the tumor site, the strata found were tumor in the rectum (66.1%), anal canal (32.1%), anal canal and rectum (0.9%) and anal margin (0.9%). Patients who presented comorbidities were 59.3% of 108 individuals assessed, such as hypertension, present in 50.9% of cases, followed by 14.8% of patients who had Diabetes Mellitus. It is pointed out that 38.4% reported diarrhea during treatment.

Regarding the prevalence of radiodermatitis, only one patient did not present this event. The percentage distribution for the severity grades was 0.9%, 31.3%, 33.0%, 33.9%, and 0.9% for the 0, 1, 2, 3, and 4 Grades, respectively. The overall prevalence rate of radiodermatitis was 99.1%, with 34.8% in the most severe grades (3 and 4). Of the 39 patients who developed more severe grades of radiodermatitis, 36 were during the first phase of treatment, from the 12th to the 30th fraction, and the remaining cases, during reinforcement.

Table 1 data show the association of sociodemographic and clinical variables with the outcome radiodermatitis.

**Table 1.** Association between sociodemographic and clinical variables and radiodermatitis grade – Rio de Janeiro, RJ, Brazil, 2018.

	Radiodermatitis grades					
Variables	Grades 0–2		Grades 3–4		OR (95% CI)*	p**
	n	%	n	%		
**Sex**						
Male	35	77.8	10	22.2	1.0	
Female	38	56.7	29	43.3	**2.60 (1.05–6.36)**	**0.038**
**Age**						
Up to 64 years	43	75.4	14	24.6	1.0	
65 years and older	30	54.5	25	45.5	**2.56 (1.15–5.72)**	**0.020**
**Education**						
Educated	71	66.4	36	33.6	1.0	
Uneducated	2	40.0	3	60.0	2.96 (0.47–18.51)	0.227
**Skin color**						
White	32	72.7	12	27.3	1.0	
Non-white	41	60.3	27	39.7	1.76 (0.77–3.99)	0.177
**Marital coexistence**						
Yes	38	74.5	13	25.5	1.0	
No	35	57.4	26	42.6	2.17 (0.97–4.88)	0.058
**Smoking**						
Never smokers	41	71.9	16	28.1	1.0	
Former smokers	19	59.4	13	40.6	1.75 (0.70–4.37)	0.228
Smokers	10	58.8	7	41.2	1.79 (0.58–5.53)	0.309
**Alcohol use**						
No	38	62.3	23	37.7	1.0	
Yes	32	71.1	13	28.9	0.67 (0.29–1.53)	0.343
**Comorbidity**						
No	29	65.9	15	34.1	1.0	
Yes	42	65.6	22	34.4	1.01 (0.45–2.27)	0.976
**Tumor stage**						
1	4	57.1	3	42.9	1.0	
2	55	72.4	21	27.6	0.51 (0.11–2.47)	0.402
3	14	48.3	15	51.7	1.42 (0.27–7.54)	0.675
**Tumor site**						
Rectum	58	78.4	16	21.6	1.0	
Anal canal	15	39.5	23	60.5	**5.56 (2.37–13.06)**	**<0.0001**
**Diarrhea report**						
No	46	66.7	23	33.3	1.0	
Yes	27	62.8	16	37.2	1.19 (0.54–2.63)	0.675

* OR (95% CI) Odds Ratio and value in the 95% confidence interval; ** chi-square test.

In this Table 1, there is a higher prevalence of Grades 3 and 4 radiodermatitis in women (43.3%), who are 2.60 times more likely to develop such an event, when compared to men (p-value = 0.038), and in patients over 65 years old (45.5%), whose chance was 2.56 times higher when compared to younger ones (p-value = 0.020). One result that drew attention was that, among smokers and former smokers, the prevalence of Grade 3 and 4 radiodermatitis was higher than 40%.

Among the clinical characteristics, only tumor site was associated with the occurrence of radiodermatitis Grades 3 and 4, and patients with anal canal tumor had a prevalence of radiodermatitis Grades 3 and 4 of 60.5%, with 5.56 times more chances of developing this outcome, compared to those with rectal tumors.

Of the 53 cases of diarrhea, 37.2% developed more severe cases of radiodermatitis. Regarding tumor staging, patients with more advanced grades showed a trend of greater severity of radiodermatitis, as observed in patients who were in stage 3, with 51.7% of frequency of Grades 3 and 4.

Regarding treatment-related variables, there was a higher prevalence of radiodermatitis Grades 3 and 4 in patients exposed to cobalt, when compared to linear accelerators (p-value = 0.011), who were 3.7 times more likely to develop more severe grades of radiodermatitis. Another significant association was observed in relation to the technique used, as patients exposed to IMRT and VMAT techniques had a higher prevalence of Grades 3 and 4 radiodermatitis (44.6%), when compared to those exposed to 2D or 3D techniques, with odds of 2.42 times higher. Data are detailed in Table 2.

**Table 2.** Association between treatment-related variables and radiodermatitis grade – Rio de Janeiro, RJ, Brazil, 2018.

	Radiodermatitis grades					
Variables	Grades 0–2		Grades 3–4			
	n	%	n	%	OR (95% CI)*	p**
**Device type**						
Linear accelerator	66	70.2	28	29.8	1.0	
Cobalt	7	38.9	11	61.1	**3.70 (1.30–10.54)**	**0.011**
**Total dose**						
4,200 to 5,000 cGy	16	59.3	11	40.7	1.0	
5,400 to 6,000 cGy	57	67.1	28	32.9	0.72 (0.29–1.74)	0.459
**Technique type**						
2D or 3D	42	75.0	14	25.0	1.0	
IMRT and VMAT	31	55.4	25	44.6	**2.42 (1.09–5.40)**	**0.029**
Combined CT **						
No	12	70.6	5	29.4	1.0	
Yes	58	63.0	34	37.0	1.41 (0.46–4.34)	0.551

*OR (95% CI) Odds Ratio and value in the 95% confidence interval; ** chi-square test. cGy – absorbed dose unit submultiple; CT – chemotherapy; IMRT – Intensity-Modulated Radiation Therapy; VMAT – Volumetric Modulated Arc Therapy.

In the assessment of temporary treatment interruption detailed in Table 3, 15 patients (13.4% of the sample) had treatment interruption due to the severity of radiodermatitis. It was found that, of 18 patients treated with cobalt device, 22.2% had treatment interruption, while, of 94 patients treated with linear accelerator, only 11.7% interrupted treatment. Interrupted treatment days ranged from 3 to 46 days, with a mean of 16.74.

**Table 3.** Association between temporary radiotherapy interruption and radiodermatitis grades depending on the type of treatment – Rio de Janeiro, RJ, Brazil, 2018.

	Treatment interruption					
Variables	Yes		No			
	n	%	n	%	OR (95% CI)*	p**
**Radiodermatitis grades**						
Grades 0–2***	0	–	73	100		
Grades 3–4	15	38.5	24	61.5	–	**<0.0001**
**Device type**						
Linear accelerator	11	11.7	83	88.3	1.0	
Cobalt	4	22.2	14	77.8	0.46 (0.13–1.66)	0.230

* OR (95% CI) Odds Ratio and value in the 95% confidence interval; ** chi-square test. ***It was not possible to calculate the Odds Ratio for radiodermatitis grade, as it contains a zero value.

## DISCUSSION

The data showed a high prevalence of radiodermatitis in patients with anal and rectal cancer, especially severe grades (34.8%). There was an association between the severity of radiodermatitis and RT interruption, with a mean of 16 days interrupted. These data from the present study can be compared with the literature in this specific population.

A retrospective study conducted in American institutions to assess the toxicity and efficacy of RT by IMRT reviewed the records of 148 patients who received, in association with CT, a median of 28 fractions of 51.25 Gy for anal cancer treatment. Toxicity was classified as acute (less than six months), late (more than six months), and severe, when higher than Grade 3 by RTOG or CTCAE. The most common acute toxicity was hematologic, with 41%, followed by skin, with 20%, and gastrointestinal tract, with 11%. Of the 20% of severe skin toxicity, 29 cases were Grade 3, and 1 case, Grade 4. It was concluded that IMRT with CT resulted in excellent local disease control and acceptable toxicity^(21)^.

An Italian study that retrospectively reviewed the records of 84 anal cancer patients treated with CT combined with IMRT assessed acute toxicity, early, late, overall treatment time and discontinuations, colostomy-free survival, and tumor response. Acute toxicity was assessed by the CTCAE scale, from the end of RT until six months later. Regarding treatment characterization, 55% of patients received the dose of 56Gy, 30%, less than 56Gy, and 15%, more than 56Gy, with a mean of 47.5 days of RT^(22)^.

Among the severe acute toxicities, skin toxicity was the most frequent, present in 19 of 84 patients assessed (23%), followed by gastrointestinal toxicity (5%). Treatment interruption occurred in 65 patients (77%), with a mean interruption of seven days. The main cause was skin toxicity, with 63% (41 patients)^(22)^.

The authors also assessed the association of sociodemographic characteristics and treatment with severe acute toxicity, showing the association only of treatment dose. It was concluded that treatment with IMRT showed excellent clinical results and low toxicity^(22)^.

These studies to support the discussion brought the percentages of severe skin toxicity with the use of IMRT around 20%, a value lower than that found in this research. Regarding treatment interruption, the temporary suspension for seven days mentioned in one of the studies^(22)^ was also lower than that identified. Other investigations, however, present higher values of severe radiodermatitis in patients with this type of cancer, which ranged from 46 to 50%^(23,24)^.

This is the case of a prospective study that assessed toxicity, quality of life and clinical outcomes in patients treated with concomitant IMRT and CT. Fifty-eight patients participated in the study, with a mean age of 56 years, 52% women who received a mean of 63 Gy of radiation dose for primary tumor and 58.5 Gy for enlarged nodules^(23)^.

Regarding acute toxicity, the researchers detected 53% of Grade 1 + 2 cases and 46% of Grade 3 + 4 cases on the skin; 38% of hematological toxicity; and 9% of gastrointestinal toxicity. Twenty-six patients (45%) discontinued treatment; of these, 23 (88%) were due to radiodermatitis, with an average of eight days of interruption^(23)^.

There was a correlation between received dose-volume and skin toxicity. It was thus considered that IMRT reduced hematological and gastrointestinal toxicity, compared to the 2D and 3D technique, without compromising local disease control^(23)^.

More recently, authors have stated that, although IMRT has been implemented to reduce toxicity, treatment interruptions and increase survival, robust studies on these effects are lacking. In view of this, they performed a retrospective analysis of data from 132 patients with anal squamous cell cancer treated with CT and RT with curative intent, to assess toxicities and patient survival.^(24)^.

Of the 132 patients, 70.5% were women and with a mean age of 67 years, a subgroup of 64 was studied for toxicity, with a mean treatment time of 37 days. Acute toxicity Grade 3 or higher was present in 34 patients (53%), of whom 50.7% developed dermatological toxicity, 56.2%, non-hematological, and 12.3%, gastrointestinal. Twenty-three patients (36%) had treatment interruption of one or more days^(24)^. The research concluded that IMRT reduced gastrointestinal and genitourinary toxicity compared to the conventional method, with similar patient survival rate^(24)^.

From the above, studies that integrate the discussion show that, although IMRT is effective in reducing other toxicities caused by RT, it still causes high rates of toxicity in the skin^(21–24)^. This statement is in line with the result obtained in this study, which showed that IMRT had a significant association with the severe radiodermatitis outcome.

It is also noteworthy that, although two studies^(22,23)^ have identified the irradiated dose as a predictive factor of radiodermatitis, the same was not confirmed in the results obtained. In the present investigation, the associated predictive factors were gender, age and type of device used in RT. Regarding device type, it is emphasized that the therapeutic modality teletherapy, in which there is a physical distance between patient and radiation source, can be implemented with the use of linear or cobalt accelerators^(25)^. The data showed higher chances of more severe grilles of radiodermatitis among patients who used a cobalt device.

Cobalt-60 devices use photon radiation, whose energy is 1.17 to 1.33 megaelectron volt, while linear accelerators produce X-ray beams with high energy, from 6 to 15 megaelectron volts or accelerated electrons, which allow treatments capable of delivering different doses simultaneously, in addition to protecting healthy structures close to the tumor. Thus, linear accelerators decrease the side effect for patients, as the radiation emitted reaches cancer cells that are sensitive, allowing healthy cells to recover more easily^(25)^.

As for the association with gender and age, among the risk factors of radiodermatitis are areas of skinfolds, which result in friction, heat and humidity^(7,8)^. In the case of women, the pelvis has more folds, and cleaning after eliminations causes more friction than the male. Therefore, it is inferred that this factor, added to histological changes of the skin with aging, justifies the higher prevalence of radiodermatitis in elderly women.

Another aspect to be pointed out is that the existence of monitoring of patients in the nursing consultation in studies supporting the discussion has not been described. Another inference that is made is that nursing interventions performed in the consultation in the researched scenario contributed to the achievement of better indicators of severe skin toxicity and treatment interruption, when compared to the literature.

This statement is based on research results that show the positive effect of such interventions^(26,27)^. A systematic review that assessed the effectiveness of nursing interventions to prevent and treat the side effects of RT indicated that the nursing consultation is an important intervention in improving communication with patients and their satisfaction. Moreover, it evidenced the effectiveness of using *Calendula officinalis* in radiodermatitis prevention, applied in the context of the nursing consultation^(26)^.

Another systematic review brought improvements in quality of life, from the greater provision of information, contribution to the abandonment of alcohol and smoking, reduction of depressive symptoms, improvement of compliance with RT as benefits to cancer patients of nursing interventions^(27)^.

In the nursing consultation of the studied setting, a nurse applies the Care Protocol for Radiodermatitis guidelines, which recommend for patients who undergo pelvic irradiation the use of hydrophilic cream with *Calendula officinalis* in its composition in Grades 0 and 1. In the presence of wet flaking, silver sulfadiazine 1% is used in the injury. In case of report of diarrhea classified as Grade 2 or higher on the RTOG scale, and with observation in the physical examination of intact skin in the irradiated area, the protocol recommends the use of a skin protectant spray once a day after RT^(20)^.

The protocol also establishes as guidelines to patients to reduce toxicities: hydration of at least two liters of liquids per day, if there is no water restriction; not ingesting alcoholic beverages during treatment; not smoking during RT; not using chemicals on the skin before RT, such as perfumes, talc, corn starch, creams and others; not using the force of water jet on irradiated skin, washing gently and protecting the markings made in simulation; avoiding hot and prolonged baths, preferring the use of neutral or slightly acidified soap; not exposing the skin to the sun during treatment; avoiding using underwear during the night^(20)^.

Therefore, it is considered that nursing consultations enable the management of clinical indicators. Based on its interpretation by nurses based on clinical reasoning, a care plan is reviewed, with the prescription of actions aimed at preventing the progression of radiodermatitis to more severe grades and treatment interruption.

Regarding the temporary treatment interruptions, the rate was 13%, with a higher number of interrupted days than the literature, which indicated a period of around eight days. A study comparing short interruptions, in which the total time did not exceed eight days, with long interruptions, considered that short interruptions due to acute and late toxicities were acceptable, compared to long-term ones^(28)^.

However, evidence of the impacts of interruptions on treatment efficacy in this population is still limited. Therefore, The Royal College of Radiologists guideline recommends that interruptions when necessary be as short as possible^(29)^.

The study limitations were incomplete medical records, underreporting of diarrhea event classification from the RTOG scale and no sample calculation in the research, since the retrospective cut of one year was adopted for the analysis of the records of only one institution.

## CONCLUSION

Radiodermatitis had a prevalence of 99.1% in patients with anal canal and rectum cancer, with 34.8% severe grades. The sociodemographic variables female sex and age greater than 65 years, the clinical variable anal canal tumor site and treatment variables cobalt device treatment and IMRT were predictive of severe grades of radiodermatitis. Treatment interruption occurred in 13% of patients, and was associated with cases of severe radiodermatitis.

By highlighting the outstanding prevalence, predictive factors and severity of radiodermatitis in these patients, as well as its impacts, the findings point to the need for further investigations. In particular, studies that assess the risk factors of radiodermatitis in other scenarios and with more comprehensive methodological designs are pointed out, as well as that test interventions based on knowledge of these risk factors, due to the reduction in the prevalence and severity of this outcome.

## ASSOCIATE EDITOR

Thelma Leite de Aráujo
